# Population-based mechanistic modeling allows for quantitative predictions of drug responses across cell types

**DOI:** 10.1038/s41540-018-0047-2

**Published:** 2018-02-24

**Authors:** Jingqi Q. X. Gong, Eric A. Sobie

**Affiliations:** Department of Pharmacological Sciences, Icahn School of Medicine at Mount Sinai, New York, NY USA

## Abstract

Quantitative mismatches between human physiology and experimental models can be problematic for the development of effective therapeutics. When the effects of drugs on human adult cardiac electrophysiology are of interest, phenotypic differences with animal cells, and more recently stem cell-derived models, can present serious limitations. We addressed this issue through a combination of mechanistic mathematical modeling and statistical analyses. Physiological metrics were simulated in heterogeneous populations of models describing cardiac myocytes from adult ventricles and those derived from induced pluripotent stem cells (iPSC-CMs). These simulated measures were used to construct a cross-cell type regression model that predicts adult myocyte drug responses from iPSC-CM behaviors. We found that (1) quantitatively accurate predictions of responses to selective or non-selective ion channel blocking drugs could be generated based on iPSC-CM responses under multiple experimental conditions; (2) altering extracellular ion concentrations is an effective experimental perturbation for improving the model’s predictive strength; (3) the method can be extended to predict and contrast drug responses in diseased as well as healthy cells, indicating a broader application of the concept. This cross-cell type model can be of great value in drug development, and the approach, which can be applied to other fields, represents an important strategy for overcoming experimental model limitations.

## Systems pharmacology: translation of drug responses across cell types

The quantitative limitations of experimental models, which can impair the development of effective therapeutics, can be overcome through a combination of mechanistic simulations and statistical analysis. A team from Icahn School of Medicine at Mount Sinai led by Eric Sobie devised a computational method to quantitatively translate drug responses across cell types. The method involves mechanism-based simulations of heterogeneous populations combined with a multivariable regression model that translates between cell types. Simulation results presented in the study show that the response to a drug in one cell type can be predicted with quantitative accuracy from physiological recordings made in another cell type, as can differential drug responses observed in diseased compared with healthy cells. This methodology can be used in drug development to better predict clinical responses based on experiments performed in preclinical models.

## Introduction

While the goal of much biomedical research is to understand human physiology and pathophysiology, direct human experiments are often infeasible and/or unethical. Because of this, experimental models of human physiology are often required. These can include cell culture models and animal models of human disease. To the extent that they provide a reasonable representation of the human system of interest, the experimental models are useful. However, when a behavior or physiological response in the experimental model does not match the behavior or response seen in the target system, the limitations of the experimental model become a concern. These differences may be qualitative; for instance, a drug that is efficacious in a mouse model of disease may fail completely at treating people because mice and humans express different isoforms of a protein. Often, however, these differences are quantitative. For example, a diabetes drug may lower blood glucose in both a mouse model and in diabetic patients, but to different extents.

A strategy for addressing this issue is to build mathematical frameworks that correct for the limitations of the experimental model. In some cases, for instance calculating the appropriate dose of a drug by considering a patient’s weight, this is trivial. In other cases empirical correction factors can be derived through painstaking trial and error. However, such correction factors generally only apply under specific conditions, and no general method exists to quantitatively correct for the inaccuracies of how an experimental model will respond to a variety of relevant perturbations.

An example of considerable immediate importance concerns electrical activity in cardiac myocytes derived from induced pluripotent stem cells (iPSC-CMs). Because these cells are a readily obtainable and renewable source of human cardiac myocytes, they are gaining popularity as a potential platform to screen drugs for toxicity.^1,2^ The cells, however, exhibit immature physiology compared with ventricular myocytes from adult hearts,^3,4^ and it remains unclear how well drug tests performed in iPSC-CMs will recapitulate the effects observed in human hearts.

We hypothesized that population-based mechanistic simulations^5–9^ could be used to quantitatively map physiological responses between cell types. Recent years have seen the development of methods that allow for the simulation of realistic variability between individuals using heterogeneous populations of mechanistic models.^5–7^ These approaches do not only allow for variability to be reproduced—when appropriate statistical methods are applied to the simulation results, these approaches can provide insight into differences between individuals in drug responses^7,10^ and allow for the development of sample-specific models.^11,12^ To extend these ideas, we attempted to translate drug responses from iPSC-CMs to human adult ventricular myocytes. Beginning with mathematical models of two cell types,^13,14^ we combined simulations of heterogeneous populations with multivariable regression approaches. The resulting model could be used to predict, with quantitative accuracy, drug effects in human adult myocytes based on recordings in iPSC-CMs. Moreover, we found that the approach could be generalized to quantitatively predict effects in diseased myocytes and across multiple species. This strategy is practically useful to address contemporary problems in drug development, and it provides a framework for addressing the vexing question of experimental model limitations.

## Results

### **Human iPSC-CMs and adult myocytes exhibit quantitative differences in their responses to ionic current perturbations**

**Fig. 1:**
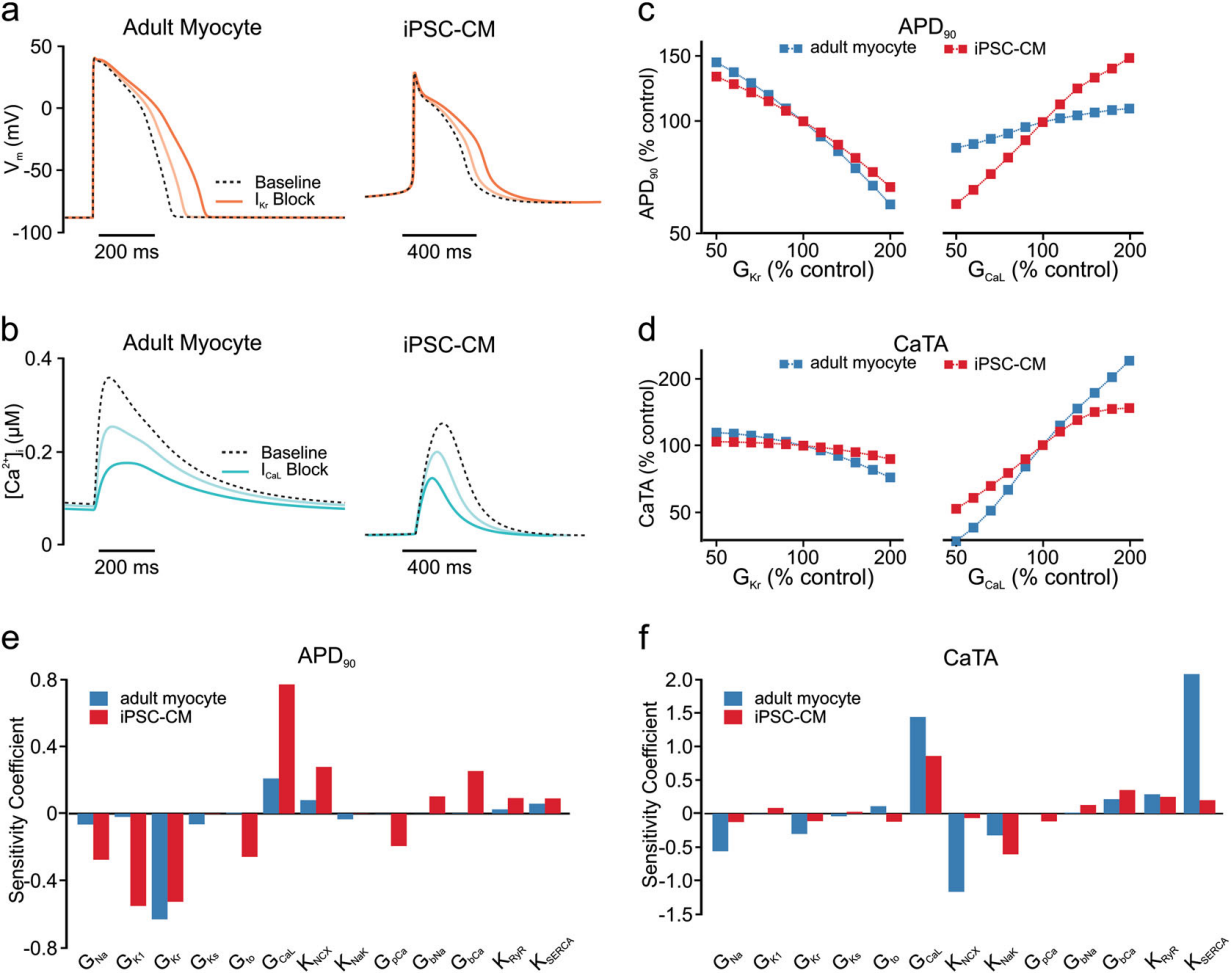
Human adult myocyte and human iPSC-CM responses to perturbations in ion transport pathways. **a** Action potential (AP) waveforms simulated in human adult myocyte and iPSC-CM mathematical models before (dashed lines) and after (solid lines) 25 and 50% block of *I*_Kr_. **b** Calcium transient (CaT) time courses of the two cell types at baseline (dashed lines), and after 25 and 50% block of *I*_CaL_ (solid lines). **c**, **d** Quantification of AP duration at 90% repolarization (APD_90_, **c**) and CaT amplitude (CaTA, **d**) as a function of maximal conductances controlling *I*_Kr_ (*G*_Kr_, left) and *I*_CaL_ (*G*_CaL_, right) in adult myocyte (blue) and iPSC-CM (red) models. All variables are expressed as a percentage of the control value obtained in the absence of perturbation. **e**, **f** Sensitivity coefficients indicating the extent to which perturbations in each ion transport pathway causes changes in APD_90_ (**e**) and CaTA (**f**). Coefficients are shown for both adult myocyte (blue) and iPSC-CM (red) models

We performed simulations to understand differences between iPSC-CMs and human adult myocytes in the electrophysiological responses to drugs. Figure 1a and b, respectively, shows how the two cell types respond to block of rapid delayed rectifier (*I*_Kr_) and L-type Ca^2+^ (*I*_CaL_) currents by 25 and 50%. The two cell types exhibit differences in baseline action potential (AP) and calcium transient (CaT) morphology, but qualitatively similar responses to ionic current blockade. We quantified the effects of ionic current perturbation by calculating the AP duration at 90% repolarization (APD_90_) and CaT amplitude (CaTA). Figure 1c and d, which plot these outputs over a range of *I*_Kr_ and *I*_CaL_ scaling factors, makes clear the quantitative divergence between human iPSC-CMs and adult myocytes.

To examine how the two cell types respond to a range of ionic current perturbations, we performed parameter sensitivity analyses^15–17^ of the two models. Across 13 ion channels, pumps, and transporters that are common to both models, we observed marked differences in how perturbations to these ion transport pathways affected APD_90_ and CaTA (Fig. 1e, f). These differences highlight that the response to a drug observed in iPSC-CMs will not necessarily match the response seen in adult myocytes, and a comprehensive method is therefore required to quantitatively translate physiological responses across cell types.

### **A multivariable regression model can translate drug responses across cell types**

**Fig. 2:**
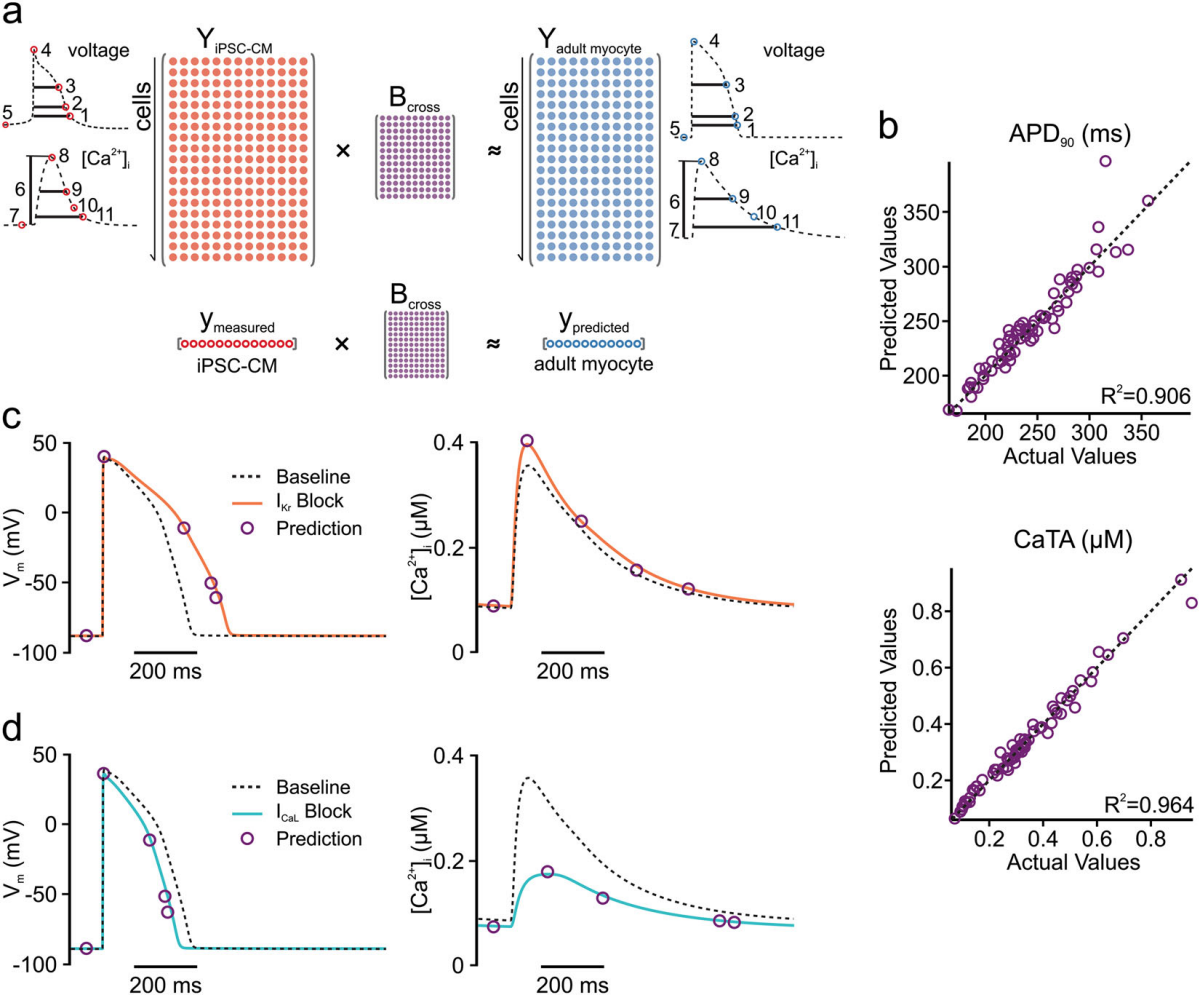
Regression model to predict adult myocyte responses from iPSC-CM physiology. **a** Upper panel, regression strategy for development of a cross-cell type model that maps physiological responses from one cell type (iPSC-CM, left) to another cell type (adult myocyte, right). Bottom panel, the use of cross-cell type model to predict drug responses with measurements from simulations or experiments. The resulting regression matrix **B**_**cross**_ serves to generate predictions on adult myocyte responses when measurements are made in iPSC-CM following the same perturbations. Insets: physiological features quantified from iPSC-CM (left) and adult myocyte (right) simulations. AP features (top): (1) AP duration (APD) at −60 mV; (2) APD at 90% repolarization (APD_90_); (3) APD at 50% repolarization (APD_50_); (4) peak membrane voltage (V_peak_); (5) resting membrane voltage (V_rest_). CaT features (bottom): (6) CaT amplitude (CaTA); (7) resting [Ca^2+^]_i_ (Ca_rest_); (8) peak [Ca^2+^]_i_ (Ca_peak_); (9) CaT duration (CaD) at 50% return to baseline (CaD_50_); (10) CaT decay time; (11) CaT duration (CaD) at 90% return to baseline (CaD_90_). For simulations of iPSC-CM spontaneous (rather than electrically paced) activity, the beating frequency was also quantified and included in the regression model. **b** Scatter plots of predictions for adult myocyte APD_90_ (top) and CaTA (bottom), with the actual values from adult myocyte simulations (abscissa) vs. the cross-cell type predictions (ordinate). For clarity, only 100 samples are shown on each of the plots, but the regression was constructed with 600 cell populations, and five-fold cross-validation was performed to calculate *R*^2^ values. **c**, **d** Adult myocyte AP and CaT responses to 50% block of *I*_Kr_ (**c**) and *I*_CaL_ (**d**). Purple circles represent regression model predictions of particular waveform features, whereas solid lines indicate numerical simulations

We developed a statistical model, based on principles of multivariable regression (Fig. 2a), to predict metrics derived from AP and CaT waveforms in adult myocytes from physiological recordings in iPSC-CMs. Similar to the approaches taken in recent studies,^5–9^ maximal conductance values for 13 ion transport pathways were randomized to generate heterogeneous in silico populations (600 cells of each type) that reflected physiological variability. A series of features were extracted from simulated time courses, including APD at several levels, diastolic and peak voltages, CaT duration at several levels, diastolic and systolic [Ca^2+^]_i_, CaTA, and spontaneous beating rate in iPSC-CMs. Partial least squares regression (PLSR)^18,19^ was then applied to the simulated population results to derive a predictive model (see Methods for details). Figure 2b shows scatter plots of APD_90_ and CaTA obtained with five-fold cross-validation. The strong correlation seen between adult model simulation results (abscissa) and regression model predictions (ordinate) indicates that the cross-cell type model is highly accurate (*R*^2^ = 0.906 for APD_90_; *R*^2^ = 0.964 for CaTA). Cross-validation results of the regression model across nine additional physiological outputs from AP and CaT are shown in Fig. S1 (Supporting Information). To confirm the model’s practical utility, we simulated 50% blockade of *I*_Kr_ and *I*_CaL_ in the baseline iPSC-CM mathematical model, then used the regression matrix **B**_**cross**_ to predict the responses to these perturbations in adult myocytes (as illustrated in Fig. 2a, bottom). The close match in Fig. 2c and d between the symbols (regression prediction) and the solid lines (adult model simulation) indicates the accuracy of the model predictions (quantified in Table S3, Supporting Information).

### **Experimental protocols that alter ionic concentrations provide critical information for the regression model**

**Fig. 3:**
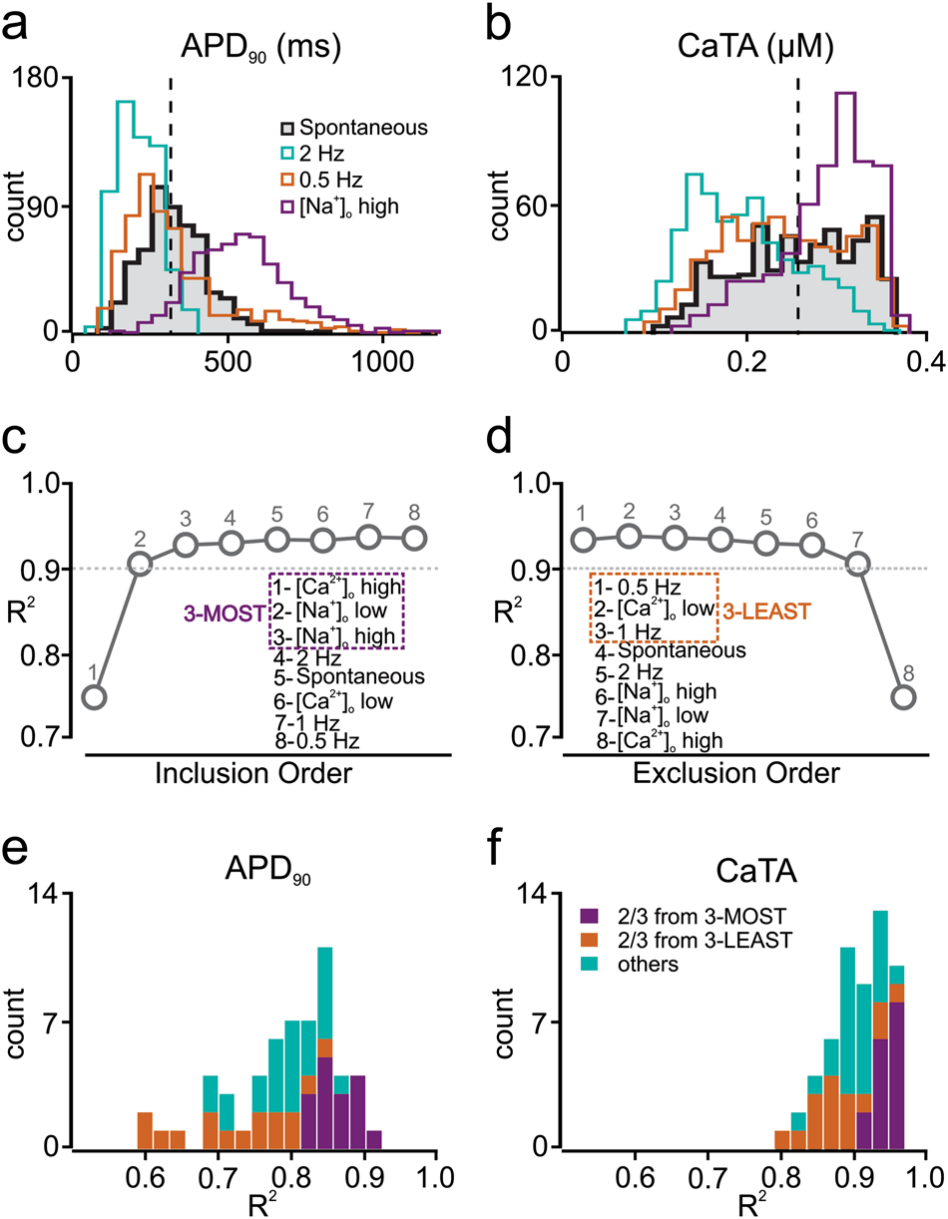
Selection of the most informative iPSC-CM simulation protocols for regression model optimization. **a**, **b** Histograms indicating how APD_90_ (**a**) and CaTA (**b**) vary across a heterogeneous population of iPSC-CMs under different simulated experimental conditions. The black, shaded histogram, representing population behavior with baseline spontaneous contraction is compared with alternative experimental conditions such as 0.5 Hz electrical stimulation (0.5 Hz, orange), 2 Hz electrical stimulation (2.0 Hz, green), and increased (300 mM) extracellular [Na^+^] ([Na^+^]_o_ high, purple). **c**, **d** Averaged *R*^2^ values across all predicted features with five-fold cross-validation, with different numbers of experimental conditions for sequential inclusion (**c**) and sequential exclusion (**d**) methods. These procedures identified the three most informative protocols (3-MOST, purple dash square, left) and the three least informative protocols (3-LEAST, orange dash square, right). **e**, **f** Distributions of adjusted *R*^2^ values for APD_90_ (**e**) and CaTA (**f**) of the 56 regression models that can be built by randomly choosing three protocols from the initial set of eight. Regression models that select two or more protocols from the 3-MOST list (purple) exhibit better predictive power than models that select two or more protocols from the 3-LEAST list (orange)

Figure 2 shows results from a regression model in which simulations performed in iPSC-CMs, under eight experimental conditions (described in Methods), are used to predict responses in adult myocytes. However, some of the eight protocols may provide partially redundant information, and it is generally unrealistic to record from an individual cell under eight separate conditions. Therefore, to prioritize efforts and direct experimental design, we evaluated the contribution of each simulated condition to the predictive strength of the cross-cell type model. First, we examined iPSC-CM population distributions of APD_90_ (Fig. 3a) and CaTA (Fig. 3b) under different experimental conditions. We observed that, compared with the baseline distributions of spontaneously beating cells (black lines and shaded histograms), some conditions caused minimal changes (e.g., 0.5 Hz pacing, orange histograms), whereas others caused more dramatic shifts in the population distributions (e.g., 2 Hz pacing, blue, and increased extracellular [Na^+^], purple). Based on these results, we hypothesized that conditions that shift these distributions are more informative and contribute more predictive power than those that cause minimal changes.

To test this hypothesis and identify the most informative simulation protocols, we constructed regression models based on sequential inclusion (Fig. 3c) or exclusion (Fig. 3d) of additional simulation conditions. With these two complementary approaches, we either included the protocol that led to the greatest improvement in *R*^2^, or excluded the protocol that caused the smallest decrease in *R*^2^ (see Methods for details). The two evaluation approaches led to similar results, and from these we concluded that the three most informative simulation conditions (3-MOST protocols) were: (1) an increase in extracellular [Ca^2+^] ([Ca^2+^]_o_ high); (2) a decrease in extracellular [Na^+^] ([Na^+^]_o_ low); and (3) an increase in extracellular [Na^+^] ([Na^+^]_o_ high). Conversely, the three least informative protocols (3-LEAST) were: (1) 0.5 Hz pacing, (2) 1 Hz pacing, and (3) a decrease in extracellular [Ca^2+^] ([Ca^2+^]_o_ low). To further validate that the 3-MOST protocols provide complementary information and improve prediction accuracy, we generated all 56 possible regression models that include 3 simulation protocols chosen from the 8, and evaluated each model based on the adjusted *R*^2^ values for APD_90_ (Fig. 3e) and CaTA (Fig. 3f). Confirming the results of the inclusion and exclusion approaches, we found that combinations with two or more protocols from the 3-MOST category (purple bars) achieved higher *R*^2^ values, compared with models that included two or more protocols from 3-LEAST (orange bars), or mixed combinations (blue bars).

Based on these results, our optimized model used in subsequent simulations was built from the three most informative experimental protocols, in addition to spontaneous beating and 2 Hz pacing. The optimized cross-cell type model achieved *R*^2^ = 0.903 for APD_90_ and *R*^2^ = 0.967 for CaTA following five-fold cross-validation.

### **Cross-cell type predictions of ionic current blockade are accurate for both selective and non-selective drugs**

**Fig. 4:**
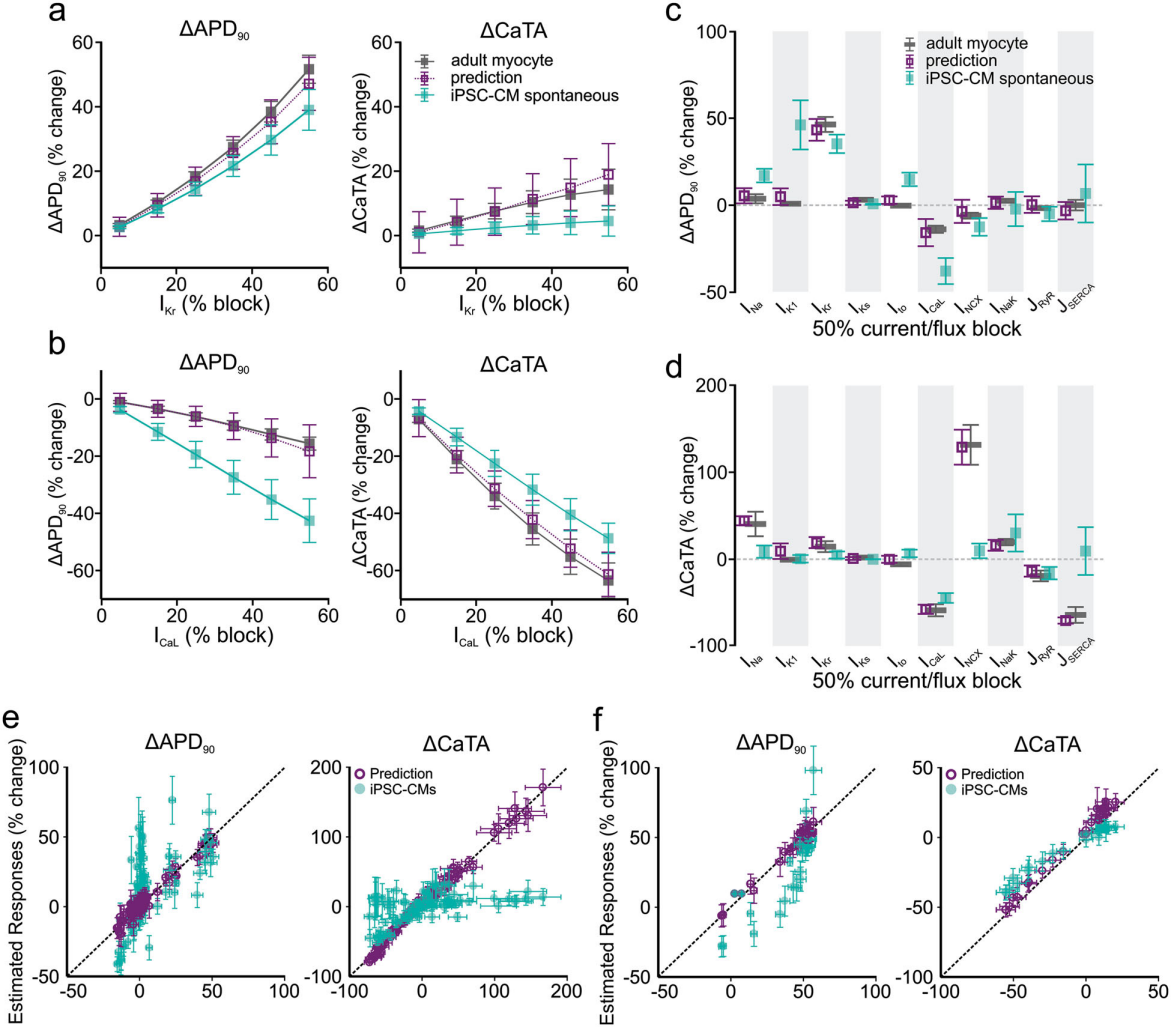
Regression model predictions of adult myocyte responses to selective and non-selective ion channel blockers. Simulations were performed in adult myocyte and iPSC-CM models to assess effects of selective (**a–d**) and non-selective ion channel blockers (**e**, **f**). In all cases, simulations were performed with heterogeneous populations of 100 cells; symbols and error bars represent mean and standard deviation, respectively**. a**, **b** Simulated selective block of *I*_Kr_ (**a**) and *I*_CaL_ (**b**) varying from 5–55% channel blockade. Responses of APD_90_ (left) and CaTA (right) are shown. In each panel, the simulated iPSC-CM response with baseline spontaneous contraction is shown in cyan, the simulated adult myocyte response is shown in dark gray, and the regression model prediction is shown in purple. **c**, **d** Simulated selective block (50%) of 10 ion transport pathways, with colors as described for **a** and **b**. Simulated drug-induced changes are presented as percent changes in APD_90_ (**c**) and CaTA (**d**). **e**, **f** Simulations were performed to assess effects of 90 hypothetical drugs that block two ion transport pathways with different potencies (**e**) and 30 real drugs that target up to five cardiac ion channels (**f**). For drug effects on APD_90_ (left) and CaTA (right), simulated adult myocyte responses (abscissa) are plotted vs. estimated responses (ordinate), either directly from iPSC-CM responses under spontaneous contraction (cyan-filled symbols) or from cross-cell type regression model predictions (purple empty symbols). Coefficient of determination (*R*^2^) was calculated to demonstrate the predictive accuracy. Taking together the 120 drugs simulated, for cross-cell type predictions, *R*^2^ = 0.9748 and 0.9858 for APD_90_ and CaTA, respectively. For iPSC-CM spontaneous responses, *R*^2^ = 0.0156 and 0.2763 for APD_90_ and CaTA, respectively

We next set out to test the ability of the optimized cross-cell type regression model to predict how adult myocytes respond to additional ionic perturbations. These simulations were performed in heterogeneous populations of iPSC-CMs and adult myocytes (100 cells in each group), which allowed us to account for variability between myocytes and estimate the precision of the predictions. Figure 4a and b shows the simulated effects of *I*_Kr_ and *I*_CaL_-blocking drugs over a range of concentrations corresponding to 5–55% channel block. Whether examining the change in APD_90_ (Fig. 4a, b, left) or the change in CaTA (Fig. 4a, b, right), the regression model prediction (purple) provides a much better estimate of the adult myocyte response (gray) than does the change in APD_90_ or CaTA observed directly from iPSC-CMs (cyan). Figure 4c and d shows predicted effects of drugs that selectively inhibit 10 ion transport pathways (all simulated at 50% block). With all drugs that cause substantial effects in adult myocytes, the cross-cell type predictions (purple) represent a much better estimation of adult myocyte effects (gray) than the straightforward iPSC-CM recordings (cyan).

Simulations were also performed to predict the effects of non-selective drugs that inhibit multiple ion transport pathways. First, for 10 important ion transport pathways (i.e., those in Fig. 4c, d), we posited 90 hypothetical drugs that blocked 2 of these pathways with different affinities. We assumed that the IC_50_ values for the primary and secondary targets differed by a factor of *e* (2.718), and we simulated drug effects at the lower IC_50_ such that the primary target was inhibited by 50% and the secondary target was inhibited by 27%. Figure 4e plots, on the abscissa, the percent changes of APD_90_ (Fig. 4e, left) and CaTA (Fig. 4e, right) simulated in adult myocytes. The ordinate represents the estimates of these values, taken either directly from spontaneously beating iPSC-CMs (cyan symbols) or calculated using the cross-cell type regression model (purple symbols). Simulations were performed in heterogeneous populations of 100 myocytes each, and mean values and population standard deviations are shown. Since, with a perfect predictor, all points would lie on the line of identity, the cross-cell type model clearly produces more accurate predictions of APD_90_ and CaTA than the iPSC-CM recordings. We also simulated 30 real drugs for which ion channel blocking effects were recently assessed in a comprehensive study.^20^ Figure 4f shows, using the same layout as Fig. 4e, that cross-cell type predictions (purple symbols) outperformed iPSC-CM spontaneous responses (cyan symbols) in predicting how these real drugs affect APs and CaTs in adult ventricular myocytes, when each drug is simulated at a concentration equal to the lowest IC_50_ value.

### **Cross-cell type regression approaches are generalizable for multiple cell types**

**Fig. 5:**
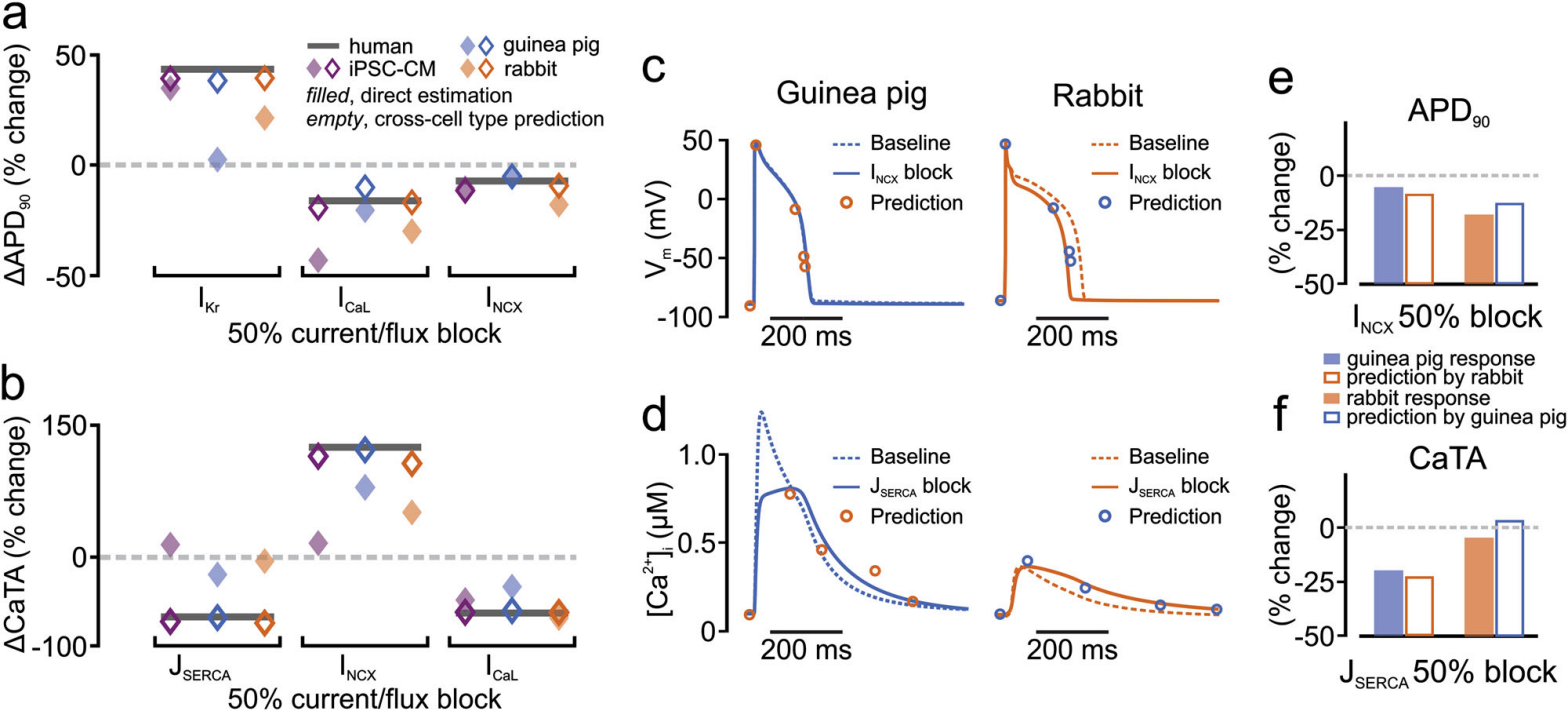
Extension of the cross-cell type regression model concept to additional cell types. **a**, **b** Adult myocyte responses to 50% current/flux block (dark gray bars) were predicted from three alternative cell types: iPSC-CM (purple symbols), guinea pig ventricular myocyte (blue symbols), rabbit ventricular myocyte (orange symbols). In each case, filled symbols represent the direct estimate from alternative cell type responses, whereas open symbols represent the cross-cell type regression model predictions. Three ion transport pathways that had large effects in adult myocytes on either APD_90_ (**a**) or CaTA (**b**) are shown. **c** Effects of 50% *I*_NCX_ block on guinea pig and rabbit ventricular action potentials. **d** Effects of 50% *J*_SERCA_ block on guinea pig and rabbit ventricular Ca^2+^ transients. In each case, baseline traces are dashed, perturbed traces are solid, and open circles represent the cross-cell type predictions of waveform features. **e**, **f** Quantification of the results shown in **c** and **d**

To determine if the cross-cell type approach was generalizable, we tested whether cross-cell type regression could be applied to additional species. Specifically, using mechanistic models of rabbit^21^ and guinea pig^22^ ventricular myocytes, we developed regression models that predict: (1) human adult myocyte drug responses from guinea pig or rabbit ventricular physiology; and (2) guinea pig myocyte drug responses from rabbit myocyte physiology, and vice versa. The overall predictive strength of these models is shown in Supplemental Fig. S3. Validations performed using 50% block of individual ion transport pathways are shown in Fig. 5. Figure 5a and b shows how responses to perturbations in human adult myocytes can be calculated from iPSC-CMs (purple symbols), guinea pig myocytes (blue symbols), or rabbit myocytes (orange symbols). The three ionic current perturbations shown are those that caused the largest changes to either APD_90_ (Fig. 5a) or CaTA (Fig. 5b) in adult myocytes. In all cases, the prediction of the cross-cell type regression model (open symbols) better approximates the adult response (black bar) compared with direct measurements performed in the alternative cell type (filled symbols). Examples in Fig. 5c–f illustrate that, when clearly different responses to a perturbation are observed in two cells, cross-cell type regression models can correct for the mismatch. For example, 50% block of NCX causes little change to APs in guinea pig myocytes (Fig. 5c, left) but shortens APs in rabbit myocytes (Fig. 5c, right). A cross-cell type regression model built from guinea pig simulations can predict the effects in rabbit, and vice versa, as quantified in Fig. 5e. As another example, 50% block of SERCA activity reduced CaTA in guinea pig myocytes (Fig. 5d, left), while causing minimal effects in rabbit myocytes (Fig. 5d, right), effects that are accurately captured by the cross-cell type models (Fig. 5f).

### **Differential drug responses in diseased vs. healthy cells can be predicted**

**Fig. 6:**
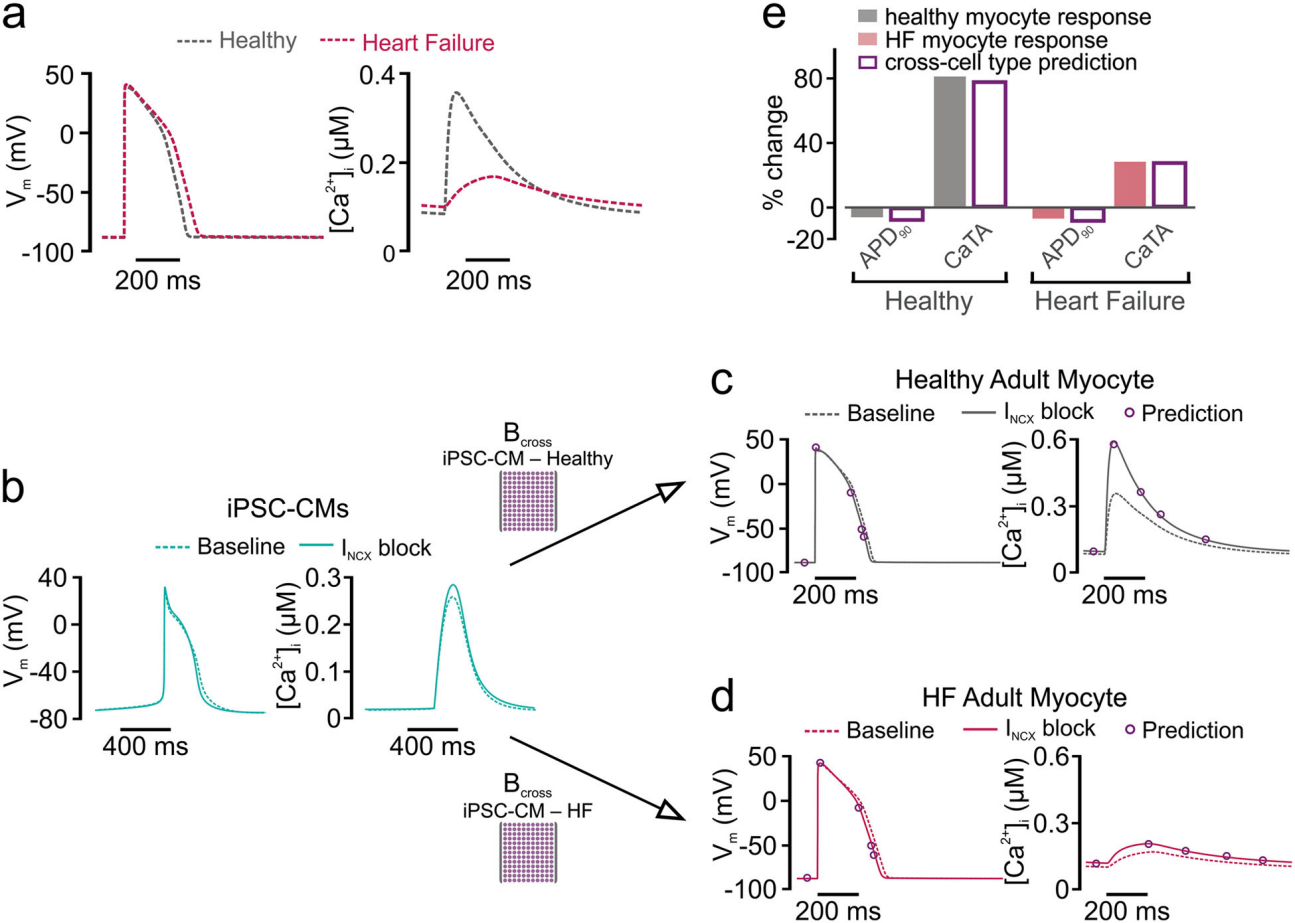
Cross-cell type modeling to predict drug responses in diseased adult myocytes. **a** Action potential (AP, left) and Ca^2+^ transient (CaT, right) simulated in the adult myocyte model, with parameters varied to reproduce a heart failure (HF) phenotype, as previously done.^23^ **b** Recordings made in iPSC-CMs can be used to predict drug responses in either healthy adult (top) or failing adult (bottom) myocytes, using alternative regression models. **c** Regression model accurately predicts that block of *I*_NCX_ by 40% causes minimal AP shortening and a large increase in CaT amplitude in healthy adult myocytes. **d** Regression model accurately predicts that block of *I*_NCX_ by 40% causes minimal AP shortening and a mild increase in CaT amplitude in failing adult myocytes. **e** Quantification of the effects observed in **c** and **d**, indicating that cross-cell type model variants can accurately predict drug responses in healthy and diseases populations of adult myocytes. Filled bars represent direct simulations, gray for healthy and pale red for failing. Empty bars are regression model predictions

In a final step, we determined whether cross-cell type regression could predict differences in drug responses between healthy and diseased cells. Based on published results,^23^ a variant of the O’Hara model was constructed to reproduce molecular and physiological alterations observed in heart failure (HF). Altered parameters (see Table S5) reflected well-described changes in HF such as reduced SERCA pump activity, upregulation of NCX, and downregulation of *I*_Ks_. Figure 6a shows that the HF variant produced slightly longer APs and reduced CaTs, recapitulating the hallmark phenotype of HF myocytes. After generation of a heterogeneous population of HF myocytes (see Methods), PLSR produced a regression model to predict drug responses in HF myocytes. With this approach, as illustrated in Fig. 6b, recordings obtained in iPSC-CMs can be used to predict responses in either healthy myocytes (top) or HF myocytes (bottom), using the respective regression matrices for prediction. As an example, these models can successfully predict the differential effects on CaTs seen in healthy and HF cells when NCX is blocked by 40%. This perturbation causes a dramatic increase in CaTA in healthy myocytes (Fig. 6c), but only a small increase in HF myocytes (Fig. 6d), effects that are well predicted by the two regression models (compare symbols with solid lines). Quantification of these effects in Fig. 6e verifies the accuracy of the predictions. Thus, the results shown in Figs. 5 and 6 demonstrate that regression models can accurately translate perturbation effects across cell types, even when the direct effects of a perturbation are dramatically different between the two cell types.

## Discussion

In this study we have described a methodology combining mechanistic modeling with statistical analyses to quantitatively translate drug effects across cell types. To develop a predictive model, we first ran simulations with populations of mechanistic cardiac myocyte models. We then performed multivariable regression on the population simulation results—this regression model allowed us to translate drug responses from iPSC-CMs to human adult myocytes. The model was highly predictive, with cross-validation *R*^2^ values of 0.906 and 0.964 for APD_90_ and CaTA, respectively (Fig. 2b). Moreover, when selective and non-selective blockers of several important ion transport pathways were simulated in iPSC-CMs under multiple conditions, the cross-cell type model predicted adult myocyte responses with quantitative precision. These predictions greatly outperformed a naive approach in which adult myocyte drug responses were assumed to be identical to those seen in iPSC-CMs (Fig. 4). Importantly, accurate predictions were also obtained when the strategy was applied to ventricular myocyte models from additional species and to a model of diseased human myocytes (Figs. 5, 6), demonstrating the general utility of the concept.

This novel cross-cell type regression model can address, in a quantitatively rigorous way, differences between an experimental model and the system that is ultimately of interest. The prediction model developed in this study possesses tremendous potential as a practical tool for toxicity testing in drug development, and the overall strategy, which can readily be applied to other fields of research, offers a roadmap for overcoming the limitations that are inherent to experimental models.

### **The potential impact of cross-cell type modeling in drug development**

Human iPSC-CMs hold considerable promise as a screening platform for assessing how drugs cause either beneficial or deleterious cardiac effects. The application that is most well developed at present is the use of iPSC-CMs for assessment of drug-induced arrhythmia risk. Specifically, studies with iPSC-CMs are a major pillar of the Comprehensive in vitro Proarrhythmia Assay, or CiPA, a public/private partnership involving the Food and Drug Administration, several pharmaceutical companies, and academic research groups.^24–26^ The goal of CiPA is to replace current pro-arrhythmia tests, which can be non-specific, with a fully in vitro assay involving ionic current measurements, mathematical modeling, and iPSC-CM experiments. A worrisome and unresolved issue within CiPA, however, is the fact that iPSC-CM physiology differs from that of adult myocytes. In general, iPSC-CMs are considered to possess an immature phenotype that more closely resembles fetal than adult heart cells.^3,4^ This is seen both structurally, in the fact that iPSC-CMs lack transverse tubules and a regular organization of sarcomeres,^27^ and functionally, in features such as the contribution of sarcoplasmic reticulum Ca^2+^ release^3^ and the densities of particular K^+^ channels.^28^

Because of these physiological differences, drug responses observed in iPSC-CMs and adult myocytes are also expected to diverge.^29–31^ The sensitivity analysis shown in Fig. 1e, f illustrates these differences by quantifying how changes in any of the shared ion transport pathways influence APD and CaTA in the two model cells. For some effects, such as changes in APD resulting from altered *I*_Kr_, the two models are largely convergent. For others, however, such as alterations in *I*_CaL_, dramatic differences are observed. Given that the L-type Ca^2+^ channel is a major ion channel in the CiPA initiative, these discrepancies present a potentially serious problem for the use of iPSC-CMs in drug development. The cross-cell type regression approach described in this study illustrates how these limitations may be overcome. Instead of approximating drug effects directly—i.e., assuming that drug-induced physiological changes are identical in adult myocytes and iPSC-CMs—a combination of mechanistic modeling and statistical analysis can be used to develop cross-cell type prediction models, and these in turn can correct for mismatches and provide more accurate predictions.

Of course, another approach to addressing differences between iPSC-CMs and adult myocytes is to improve the iPSC-CM experimental model such that the cell types become more similar. Indeed, recent years have seen many advances to optimize differentiation and produce more mature iPSC-CMs.^32–34^ We note, however, that our strategy is not in competition with these important efforts, instead it is a complementary approach that is likely to become even more urgently needed as maturation methods develop. For one thing, even if a truly optimal iPSC-CM maturation protocol can eventually be identified, cells will almost certainly continue to exhibit some differences with adult cells, and methods to understand these differences quantitatively will still be required. Additionally, the next several years are likely to see a proliferation of alternative methods rather than rapid agreement on a protocol of choice. In this scenario, the field will require ways to translate effects between different iPSC-CM production methods. Finally, a close approximation of the healthy adult ventricular myocyte is not the only cell type that is needed in drug development. Because diseased hearts and healthy hearts may respond differently to the same drugs, we also require methods to understand how therapeutics may affect particular patient populations. Figure 6 illustrates that, once appropriate regression models have been developed, recordings obtained in a single iPSC-CM preparation can simultaneously predict drug effects in both healthy and failing adult myocytes. Thus, efforts to improve the iPSC-CM experimental model will become more powerful when they are coupled to modeling approaches that can quantitatively synthesize results across different preparations.

### **Practical considerations in employing cross-cell type predictions**

In addition to developing a model that would be useful for drug development, we also addressed practical considerations required to implement such a cross-cell type approach. One of the principles underlying our model is that studying multiple experimental conditions provides additional information. In other words, simulating only a population of spontaneously contracting iPSC-CMs might not be sufficient to accurately predict drug responses in adult myocytes, but if the same iPSC-CMs are simulated under different pacing protocols, different ionic conditions, etc., then an accurate regression model can be generated. Because it is not always feasible to examine individual cells under a wide range of experimental conditions, however, we identified the most informative simulation conditions in iPSC-CMs for accurate predictions of adult myocyte responses (Fig. 3). An interesting result to arise from this analysis, which ranked experimental protocols from the most to the least informative, was that altering extracellular ion concentrations appears to be more useful than electrical pacing, a prediction that remains to be tested.

With these considerations in mind, we can contemplate how a predictive cross-cell type model can be applied in practice using experimental data. First, population simulations performed in two cell types of interest, such as those we have presented here, are used to identify the informative experimental conditions and calculate the regression matrix **B**_**cross**_. Second, experimental recordings are performed in the source cell type (e.g., iPSC-CMs), under multiple conditions, in the presence and the absence of a drug. Third, metrics are extracted from these recordings to populate a vector of features that quantify drug-induced changes in physiology. Importantly, a variety of measures must be calculated from voltage and Ca^2+^ time courses; it will not be sufficient to only use simple metrics such as APD_90_. Finally, the vector of features is multiplied by **B**_**cross**_ to predict how the drug will affect the target cell type (e.g., human adult myocytes).

In implementing an approach such as we have outlined here, an additional practical consideration that must be addressed is the mathematical model of the iPSC-CM. Although many alternative mathematical models of animal myocytes^35,36^ and human adult ventricular myocytes^37,38^ have been published, the Paci et al. model^14^ remains, at the present time, the only model of the iPSC-CM. The preferred approach to modeling iPSC-CM physiology will certainly be modified as additional data become available, and as iPSC-CMs developed under particular conditions become more completely characterized. To facilitate the future development of model improvements, an efficient and valuable approach is to choose parameters that match physiological responses (e.g., APs and CaTs) not only under baseline conditions, but in response to perturbations such as augmenting or inhibiting particular ionic currents.^11,12^ Compared with the alternative strategy of characterizing each important ion transport mechanism in every cell type of interest (i.e., fitting models to voltage clamp data), this approach offers the possibility of more rapidly tuning a model to recapitulate experimental data, once the general model structure (i.e., which ion transport mechanisms should be included) is understood.^39^ Importantly, these recent studies provide a guide for how to continually improve the cross-cell type approach as additional data are obtained and models of the relevant cell types become more advanced.

### **Extension of the cross-cell type concept to additional contexts**

Results such as those shown in Figs. 5 and 6 can initially seem quite surprising: if blocking a particular pathway causes minimal effects in one cell type, how can the model successfully reproduce more dramatic effects in another cell type? The answer to this question arises from two important aspects of the cross-cell type regression approach: (1) variability is universally imposed on all the major ion transport pathways when generating the heterogeneous cell populations; and (2) a wide range of simulation results, obtained under multiple experimental conditions, are recorded. These ensure that the underlying statistical associations are robust enough to make accurate predictions, even in cases where obvious differences exist between the source and target cells. Put another way, APD in the adult human myocyte is not simply a function of APD in the iPSC-CM; instead this output depends on an appropriately weighted average of many physiological metrics that can be measured in iPSC-CMs. Our approach therefore advocates for considering all relevant information systematically, and synthesizing based on rigorous statistical analysis, rather than focusing on particular measurements that are deemed to be most important.

Beyond being a practically useful platform, the cross-cell type regression model can also help to guide future mechanistic studies. Although the extension to rabbit and guinea pig ventricular myocyte models demonstrated that the concept is generalizable (Fig. 5), it should be noted that the prediction accuracy was not identical for all models generated. For instance, the worst-performing model, the translation from rabbit to guinea pig myocytes (Fig. S3), probably reflects important differences between the two cell types, a question that should be investigated mechanistically. The difficulty in predicting guinea pig drug responses from rabbit physiology could result from the lack of transient outward K^+^ current (*I*_to_) in the former species compared with its prominent role in the latter, and this idea could be addressed in simulations and experiments by either blocking *I*_to_ in rabbit or adding *I*_to_ in guinea pig cells to determine if their drug responses become more similar. Mechanistic insight could also be gained by examining individual simulated cells that are poorly predicted by the regression model. It is likely that specific parameter alterations in these cells cause non-linear physiological changes that prevent the linear regression model from accurately predicting these particular cells.

More broadly, the extension to additional cardiac myocyte models suggests ways that the concept can be further generalized and expanded. The number of cell types seen in the nervous system, for instance, dwarfs what is observed in the heart. However, because different types of neurons employ many of the same channels and receptors to shape their behaviors, an approach such as ours should be feasible for predicting differences. Similarly, the pathways that control processes such as cell division and apoptosis are largely shared between different cell types, although the ways that cells respond to physiological or pharmacological stimuli can be extremely different. For issues such as these, where robust mathematical models already exist,^40–45^ a cross-cell type approach such as we have outlined can be used to predict how behaviors observed in one cell type may or may not be reproduced in a related cell type.

### **Study limitations**

Although the results presented in this study are encouraging and provide confidence that the concept can be of practical use, several limitations should be noted. First, in generating the heterogeneous populations, we only varied model parameters controlling ionic current magnitudes, leaving the channel kinetics unaltered. As a result, we could only simulate drug effects through a simple pore block model. Many drugs, however, bind to ion channels in a state-dependent manner, producing effects that our approach could potentially fail to predict. These complexities could be included through the use of more complex Markov models, which have been developed for many critical channels.^46–48^ For these predictions to be accurate, however, the same kinetic scheme and drug binding characteristics would have to be used in both the source and target cell models, which is not generally the case at present. Second, although the regression model performed extremely well with simulated data, the experimental validity of these predictions remains to be tested. Although the ultimate goal of many cardiac experimental models is to predict behaviors in adult human hearts and myocytes, the difficulty of obtaining such tissue for experiments precluded the use of such tests in this study. However, alternative methods for testing the robustness of the concept do exist, for instance by testing the physiological responses to drug treatments across different iPSC-CM preparations. Studies have shown, for instance, that physiological differences exist between iPSC-CMs purchased from alternative vendors.^49,50^ These alternative iPSC-CM variants may prove a useful platform for experimentally testing this approach, and the results obtained in such a comparison may help to drive convergence toward a more optimal iPSC-CM experimental model.

## Conclusions

We developed a novel methodology that serves to translate drug effects across cell types with quantitative accuracy, an approach that is likely to be widely useful for addressing differences between experimental models and target systems of interest. Not only is the initial test case we presented, the translation from iPSC-CMs to human adult myocytes, potentially of practical use during toxicity testing to identify pro-arrhythmic drugs, the approach we have outlined can be used to streamline and optimize experimental design. Most important, the methodology we have outlined can easily be applied to other fields in which mechanistic models are well developed, potentially greatly increasing the impact of the concept and approach.

## Methods

### **Mathematical models**

We developed regression models to predict effects across cell types by using simulations performed with four cardiac myocyte models: (1) O’Hara et al. human adult ventricular myocyte model,^13^ (2) Paci et al. human iPSC-CM model,^14^ (3) Livshitz et al. guinea pig ventricular myocyte model,^22^ and (4) Shannon et al. rabbit ventricular myocyte model.^21^ O’Hara et al. simulations were performed with the model’s endocardial variant, and Paci et al. simulations were performed with the ventricular-like variant. Simulations with the O’Hara et al. model were also performed using a model variant in which parameters were altered, based on prior work,^23^ to reproduce molecular and physiological changes observed in HF. Parameter modifications made to produce the O’Hara HF variant are shown in Supplementary Table S5.

Each model, consisting of a system of ordinary differential equations, was implemented in MATLAB version R2016b (The MathWorks, Natick, MA). Differential equations were numerically integrated using MATLAB’s ode15s function, a solver for stiff systems, and computations were performed in a Windows 10 environment.

### **Simulations of heterogeneous model populations**

To generate heterogeneous populations of models, parameters controlling maximal rates of ion transport were multiplied by scale factors randomly selected from log-normal distributions. Randomly varied model parameters included: fast Na^+^ current (*G*_Na_), inward rectifier K^+^ current (*G*_K1_), rapid and slow delayed rectifier K^+^ currents (*G*_Kr_ and *G*_Ks_), transient outward K^+^ current (*G*_to_), L-type Ca^2+^ current (*G*_CaL_), Na^+^–Ca^2+^ exchanger (*K*_NCX_), Na^+^–K^+^ pump (*K*_NaK_), sarcolemmal Ca^2+^ pump (*G*_pCa_), background Na^+^ and Ca^2+^ currents (*G*_bNa_ and *G*_bCa_), sarcoplasmic reticulum Ca^2+^ release flux through ryanodine receptors (*K*_RyR_), and sarcoplasmic reticulum Ca^2+^ uptake via SERCA pumps (*K*_SERCA_). Baseline values for these model parameters can be found in Supplementary Tables S1–S2. As a result of this scaling, each individual cell in the population has a distinct profile of ion channel/pump/transporter expression levels.

Scale factors were log-normally distributed such that log-transformed values had a mean of zero and a standard deviation of 0.2624. As a result, 95% of the cells in the populations had expression levels ranging between 60 and 167% of control values. The same set of scale factors was applied to the two cell types used to construct cross-cell type regression models. Simulations were performed for every cell in the population, and physiological features extracted from the AP and CaT waveforms are as follows (also indicated in Fig. 2, inset). AP features computed were: (1) AP duration (APD) at −60 mV; (2) APD at 90% repolarization (APD_90_); (3) APD at 50% repolarization (APD_50_); (4) peak membrane voltage (*V*_peak_); (5) resting membrane voltage (*V*_rest_). CaT features computed were: (6) CaT amplitude (CaTA); (7) resting [Ca^2+^]_i_ (Ca_rest_); (8) peak [Ca^2+^]_i_ (Ca_peak_); (9) CaT duration (CaD) at 50% return to baseline (CaD_50_); (10) CaT decay time; (11) CaT duration (CaD) at 90% return to baseline (CaD_90_). Decay time was calculated as the duration from the peak of the Ca^2+^ transient to the time when the [Ca^2+^] level had been reduced by a factor of *e*. For simulations of iPSC-CM spontaneous (rather than electrically stimulated) activity, the beating frequency was also quantified.

### **Simulation protocols**

Cells that do not contract spontaneously (adult human, rabbit, and guinea pig myocytes) were electrically stimulated at 1 Hz for 120 s, which was sufficient to reach steady state in over 95% of the cells in the population. The last AP and CaT in the sequence were recorded. For spontaneously contracting iPSC-CMs, the last AP and CaT in a 120 s simulation period were recorded. Additional experimental protocols were simulated in iPSC-CMs, rabbit myocytes, and guinea pig myocytes. These included: (1) electrical pacing at 0.5 and 2 Hz for 120 s; (2) increasing and decreasing extracellular [Ca^2+^] (baseline = 1.8 mM, high = 3.0 mM, low = 0.9 mM); (3) increasing and decreasing extracellular [Na^+^] (baseline = 151 mM, high = 300 mM, low = 70 mM); and (4) increasing and decreasing extracellular [K^+^] (baseline = 5.4 mM, high = 10 mM, low = 3 mM). For the protocols that varied extracellular ion concentrations, guinea pig and rabbit myocytes were electrically stimulated, whereas spontaneously contracting iPSC-CMs were simulated. We chose these protocols because of the ease with which they can be implemented experimentally.

### **Construction and validation of cross-cell type models**

PLSR^18,19^ was used to quantitatively relate physiological features in one cell type (the source cell) to those in another cell type (the target cell). To develop these regression models, features quantified from AP and CaT waveforms (see Fig. 2, inset) were placed into matrices. The “input” matrix consisted of features simulated under multiple conditions from the cells in the source population (e.g., iPSC-CMs), whereas the “output” matrix consisted of features in the target cells (e.g., adult human myocytes). Each row in these matrices corresponded to a different cell in the population; each column corresponded to a different simulated feature. PLSR was then applied to derive a matrix, **B**_**cross**_, that translates from one cell type to another and can subsequently be used for predictions. For instance, if recordings are made experimentally in iPSC-CMs before and after application of a drug, then the drug-induced changes to the physiological features can be placed in a vector **y**_**measured**_ (Fig. 2a, bottom). This vector can then be multiplied by **B**_**cross**_ to predict the effects of the same drug in adult myocytes.

During model construction, we performed cross-validation to evaluate the prediction accuracy. For example, to perform five-fold cross-validation, populations of 600 cells were divided into five subgroups of 120 cells each. Each regression matrix **B**_**cross**_ derived from four subgroups (480 cells) was then used to predict behavior in the remaining subgroup. The performance of the cross-cell type model was evaluated using Predicted Residual Sum of Squares, or PRESS (see Supplementary Methods for details).

PLSR is an iterative approach that requires using weighted combinations of input variables (similar to principal components). PLSR is well suited for data sets in which the columns in the input matrix are correlated; however, it is important to terminate the iterative PLSR algorithm at the appropriate time to avoid overfitting.^51,52^ As described in more detail in Supplementary Methods, we chose the number of components of the PLSR model by iteratively determining the smallest number of components that minimized PRESS while simultaneously achieving a high *R*^2^ value.

### **Identification of the most informative and least informative experimental conditions**

To determine whether cross-cell type modeling was feasible, we initially constructed a model using simulated results obtained under many experimental conditions. To make this model more suitable for experimental testing, we determined the most informative experimental conditions using sequential inclusion and exclusion methods. First, although we initially simulated 10 experimental conditions (see above, simulation protocols), we found that under some conditions, more than 25% of the cells in the population exhibited abnormal dynamics (e.g., afterdepolarizations, failure to repolarize). These were excluded from the determination of the most informative conditions. Specifically, in the current study for adult myocyte and iPSC-CM simulations, both increasing or decreasing extracellular [K^+^] were excluded, resulting in eight conditions for subsequent analysis.

For the sequential inclusion method, we generated PLSR models using each of the eight experimental conditions individually, and selected the one with the highest average *R*^2^ with five-fold cross-validation. We then added each of the seven remaining conditions in turn, and selected the one that caused the largest increase in *R*^2^. This process continued until all eight experimental conditions had been ranked. Similar criteria were used for the sequential exclusion method, during which we started with all eight conditions, excluded one condition at a time, then rejected the one that whose exclusion caused the smallest decrease in *R*^2^ value.

### **Simulations of selective and non-selective drugs in heterogeneous populations**

We simulated selective and non-selective blockers of ion transport pathways by scaling the model parameters controlling the magnitudes of ionic current or flux. For example, when simulating selective blockade of *I*_Kr_ by 50%, we scaled the conductance *G*_Kr_ to 50% of its control value. For details of all tested ion transport pathways, see Supplementary Tables S3–S4.

The 90 non-selective hypothetical drugs were designed such that, of the 10 important ion transport pathways (as tested with selective blockers in Fig. 4), each hypothetical drug targets 2 of the 10 pathways with different affinities, and the IC_50_ values for primary and secondary targets differ by a factor of *e*.

To simulate the effects of non-selective drugs at a drug concentration [*C*], we scaled the model parameters controlling the maximal magnitude of ion current/flux for primary and secondary targets according to the equation:

$$\frac{{G_{\rm drug}}}{{G_{\rm no\, drug}}} = \frac{{IC_{50}}}{{IC_{50} + [C]}}$$

For the 90 hypothetical non-selective blockers, we simulated drug effects at the lower IC_50_, a concentration at which the primary target was inhibited by 50% and the secondary target was inhibited by 27%. For the 30 drugs recently characterized^20^ that target up to 5 ion channels with different affinities, we simulated drug effects at the lowest IC_50_ value such that the primary target was inhibited by 50%, whereas other channels were inhibited to lesser extents.

### **Code and data availability**

An implementation of the cross-cell type modeling approach is available at https://github.com/JQXGong/cross-cell-type-regression.git. The repository contains implementations of the mathematical models used in the study, a sample simulated data set, and customized scripts to generate a cross-cell type regression model. Code is written in MATLAB.

## Electronic supplementary material

Supplemental Methods

Supplemental Figures

Supplemental Tables

## Electronic supplementary material

**Supplementary information** accompanies the paper on the *npj Systems Biology and Applications* website (10.1038/s41540-018-0047-2).
