# Supplementary material for: Population-based mechanistic modeling allows for quantitative predictions of drug responses across cell types
Source: NPJ Syst Biol Appl. 2018 Feb 24;4:11. doi: 10.1038/s41540-018-0047-2 (PMC5825396; doi:10.1038/s41540-018-0047-2)
Supplement: Supplementary file 1 — Supplemental Methods [file 41540_2018_47_MOESM1_ESM.pdf]

SUPPLEMENTARY METHODS

Predicted Residual Sum of Squares (PRESS)

PRESS represents a statistic describing lack of prediction accuracy[1,2]. Following cross-validation, a matrix $\hat{\mathbf{y}}$ of out-of-sample predictions will be generated. Corresponding to the original **Y** matrix, for each column PRESS can be computed respectively, as:

$$PRESS = \sum_{i=1}^{n} (\mathbf{Y}_i - \hat{\mathbf{y}}_i)^2$$

While Total Sum of Square (TSS) for each column is computed as:

$$TSS = \sum_{i=1}^{n} (\mathbf{Y}_i - \bar{\mathbf{Y}}_i)^2$$

** $\bar{\mathbf{Y}}_i$ stands for the column mean of the original **Y** matrix*

All the R^2 values following cross-validation reported in the study were computed as

$$R^2 = 1 - \frac{PRESS}{TSS}$$

Decision on number of components used in PLS regression

To leave out small components that only describe noise and avoid the problems of overfitting[3,4], process for identifying the proper number of components to keep in PLS regression was implemented. In particular, we tested the number of components from 1 to the rank of the input matrix (e.g. the $Y_{iPSC\text{-}CM}$ in Fig. 2A); for every trial we computed the following statistics: 1) R^2 values from model building; 2) PRESS for predicted error following leave-one-out cross validation. The proper number of components to use was then identified so that the minimal PRESS value and a high R^2 value are achieved concomitantly.

References

1-Allen, D. M. The Relationship between Variable Selection and Data Agumentation and a Method for Prediction. Technometrics 16, 125-127 (1974).

2-Geladi, P. & Kowalski, B. R. Partial least-squares regression: a tutorial. Analytica Chimica Acta 185, 1-17 (1986).

3-Hawkins, D. M. The Problem of Overfitting. Journal of Chemical Information and Computer Sciences 44, 1-12 (2004).

4-Tetko, I. V., Livingstone, D. J. & Luik, A. I. Neural network studies. 1. Comparison of overfitting and overtraining. Journal of Chemical Information and Computer Sciences 35, 826-833 (1995).
