# Supplementary material for: Population-based mechanistic modeling allows for quantitative predictions of drug responses across cell types
Source: NPJ Syst Biol Appl. 2018 Feb 24;4:11. doi: 10.1038/s41540-018-0047-2 (PMC5825396; doi:10.1038/s41540-018-0047-2)
Supplement: Supplementary file 2 — Supplemental Figures [file 41540_2018_47_MOESM2_ESM.pdf]

SUPPLEMENTARY FIGURES

**Content in SI**	**Relevance**
Fig. S1, Fig. S2	Constructing and optimizing the cross-cell type regression model from iPSC-CMs to adult myocytes
Fig. S3	Cross-cell type regression with guinea pig and rabbit ventricular myocyte models
Fig. S4	Cross-cell type predictions of adult myocyte behavior from different mathematical models

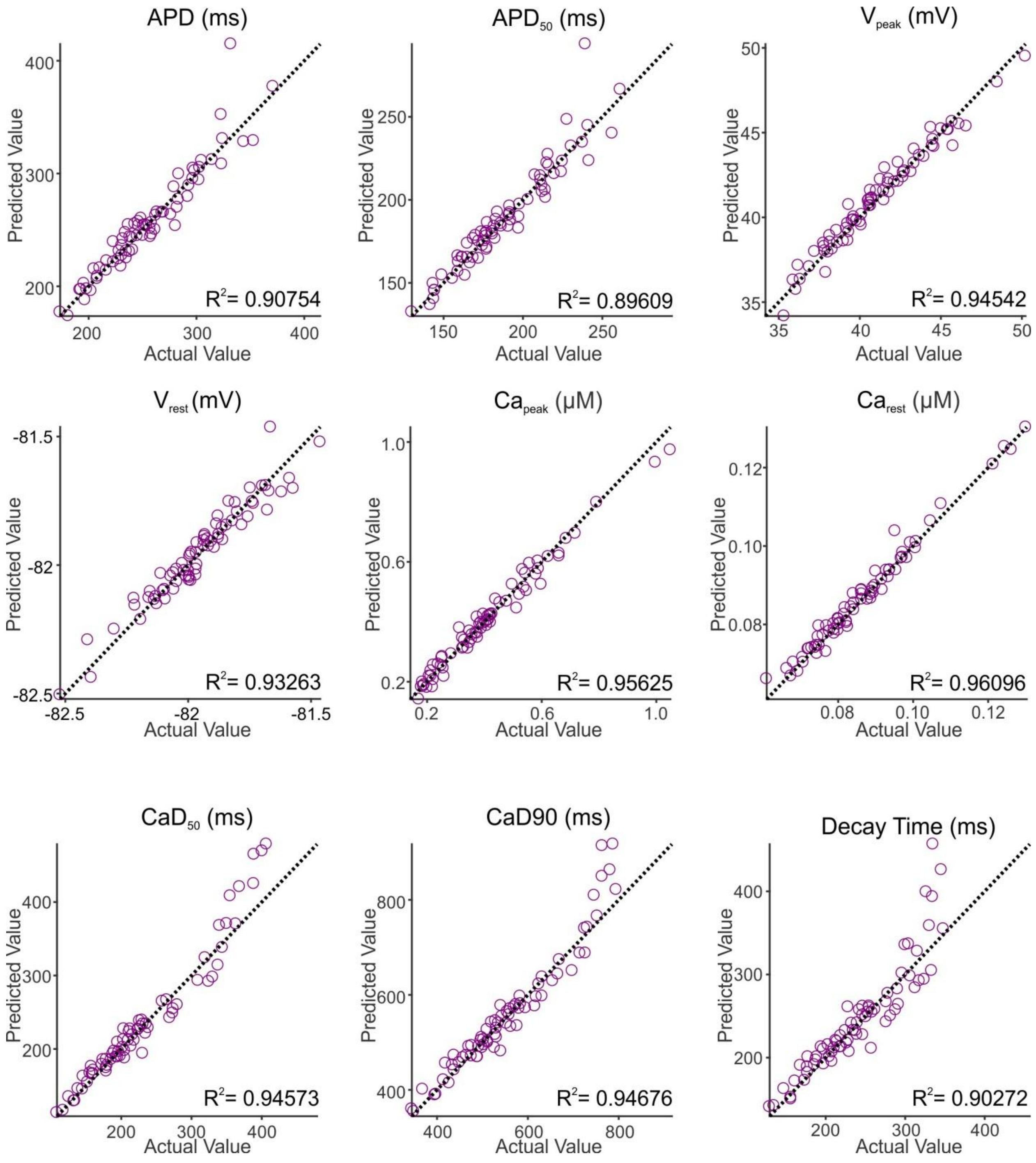

Fig. S1. Scatter plots for 9 additional physiological features (besides APD_{90} and CaTA) quantified from AP and CaT that are simultaneously predicted by the cross-cell type regression model from iPSC-CM to adult myocyte. Actual values for each feature (*abscissa*) are plotted versus predicted values (*vertical*); R^2 values were derived from five-fold cross validation. For clarity, only 100 cells are plotted although 600 cells in total were used to construct the cross-cell type regression model.

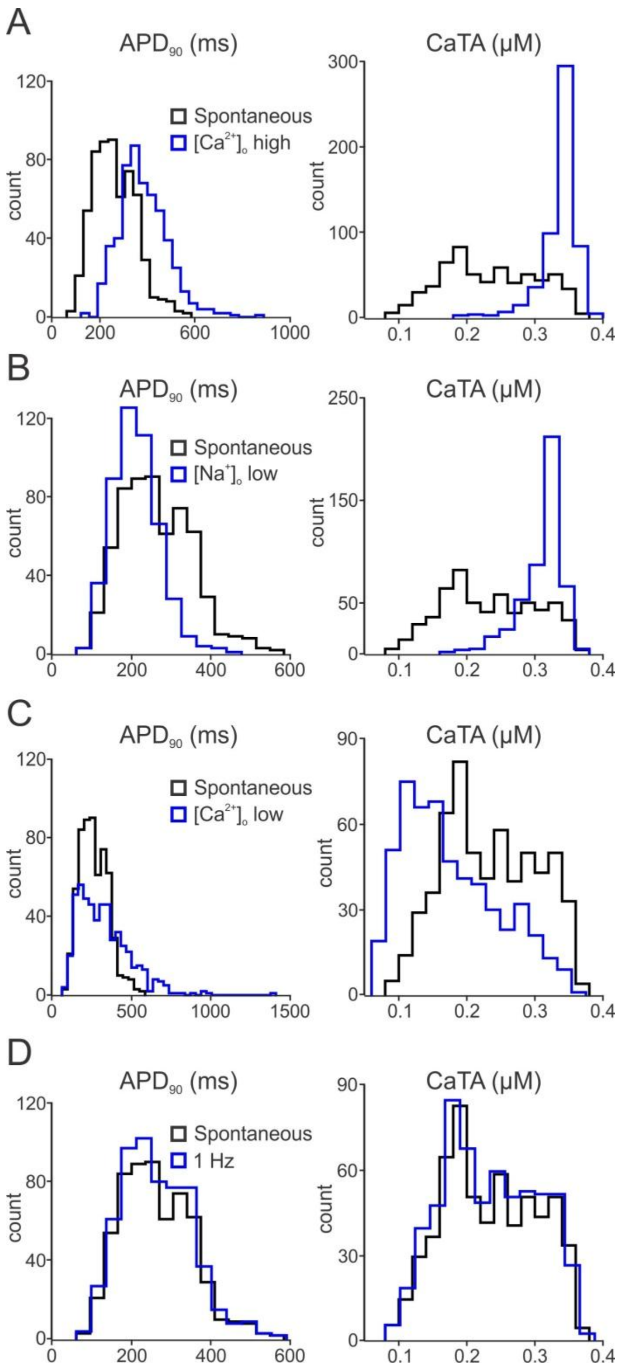

Fig. S2. Distribution of iPSC-CM population APD_{90} and CaTA of simulation results under different experimental conditions. In each plot, baseline spontaneous contraction histograms are plotted in black, and alternative experimental conditions are shown in blue. **(A)** increased extracellular Ca^{2+} (3.0 mM, $[Ca^{2+}]_o$ high); **(B)** decreased extracellular Na^+ (70 mM, $[Na^+]_o$ low); **(C)** decreased extracellular Ca^{2+} (0.9 mM, $[Ca^{2+}]_o$ low); **(D)** 1 Hz pacing (1 Hz). Note that larger shifts in APD_{90} and CaTA histograms are observed in **(A)** and **(B)** compared with **(C)** and **(D)**.

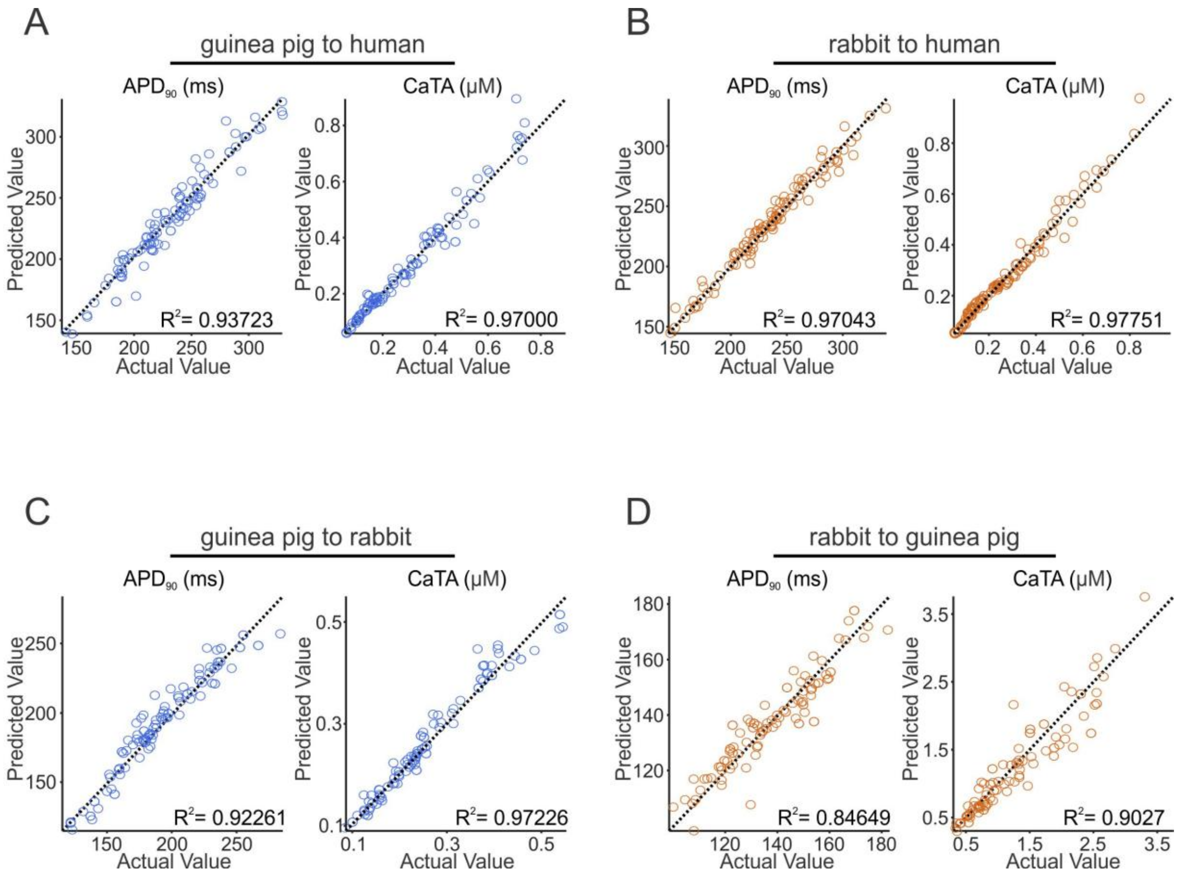

Fig. S3. Scatter plots demonstrating the predictive strength for four cross-cell type regression models: (1) guinea pig ventricular myocyte predicts human adult myocyte **(A)**; (2) rabbit ventricular myocyte predicts human adult myocyte **(B)**; (3) guinea pig ventricular myocyte predicts rabbit ventricular myocyte **(C)**; (4) rabbit ventricular myocyte predicts guinea pig ventricular myocyte **(D)**. APD_{90} and CaTA results are shown; actual values for each feature (*abscissa*) are plotted versus predicted values (*ordinate*). R^2 values were calculated following five-fold cross validation for all four regression models. For clarity, only 100 cells were plotted but in total 600 cells were used to construct the cross-cell type regression models.

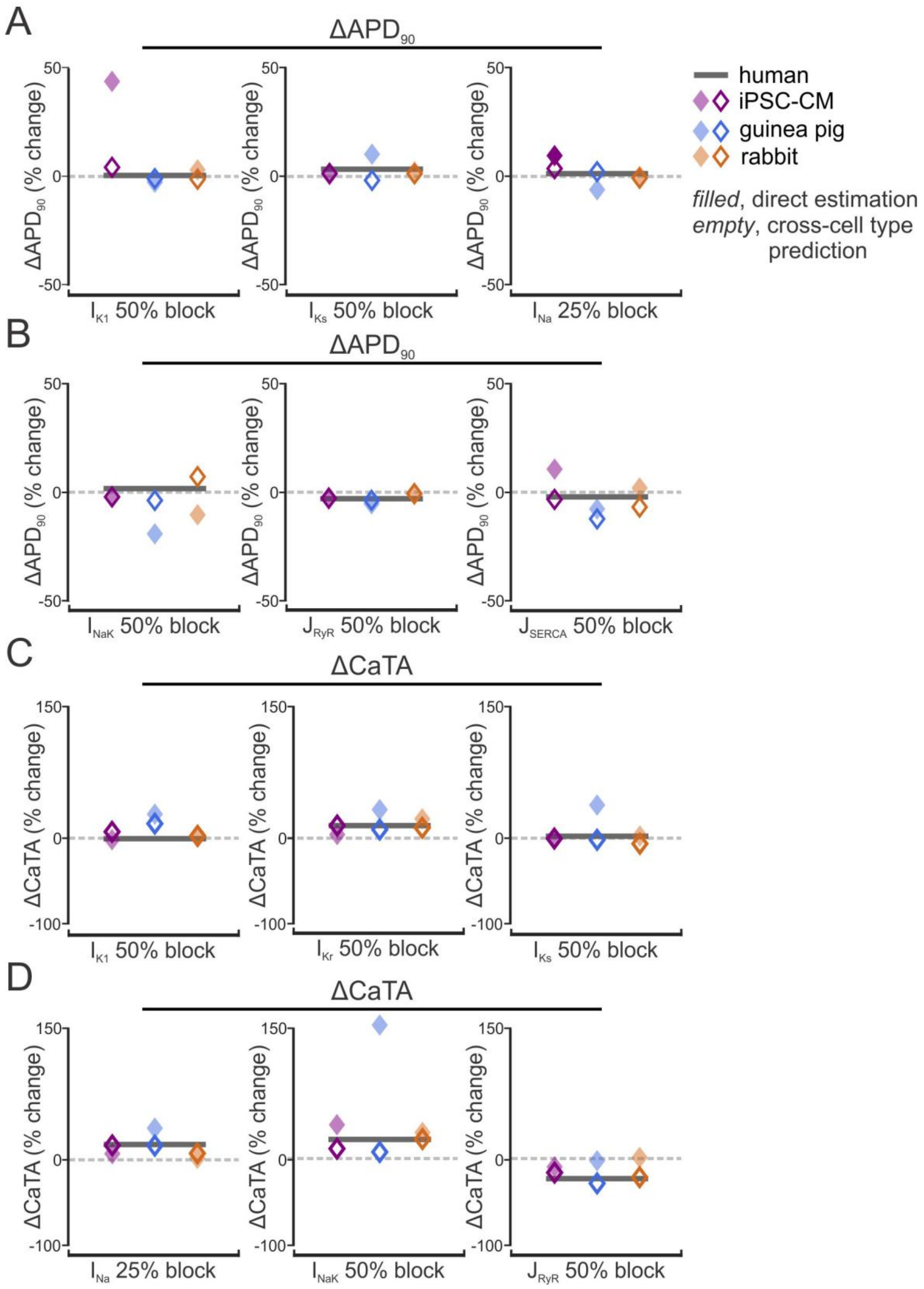
A
ΔAPD90
ΔAPD90 (% change)
50
0
-50
IK1 50% block
IKs 50% block
INa 25% block
human
iPSC-CM
guinea pig
rabbit
filled, direct estimation
empty, cross-cell type prediction
B
ΔAPD90
INaK 50% block
JRyR 50% block
JSERCA 50% block
C
ΔCaTA
ΔCaTA (% change)
150
0
-100
IK1 50% block
IKr 50% block
IKs 50% block
D
ΔCaTA
INa 25% block
INaK 50% block
JRyR 50% block

Fig. S4. Effects on APD_{90} and CaTA with simulated 50% block of ion transport pathways. Figure 6 shows the effects of blocking the three ion transport pathways that had the greatest influence on APD_{90} and CaTA. These results show the effects of blocking the remaining pathways. Simulated adult myocyte responses are plotted as dark grey bars; cross-cell type predictions originating from 3 alternative cell types are plotted as empty diamonds, and direct estimates from alternative cell type responses are plotted as filled diamonds. Consistent between iPSC-CM (purple), guinea pig ventricular myocyte (blue) and rabbit ventricular myocyte (orange) models, cross-cell type predictions achieved better accuracy in terms of capturing real adult myocyte responses. (For I_{Na}, 25% block results are shown because 50% I_{Na} blockade in the iPSC-CM model resulted in failure to initiate spontaneous action potentials).
