# Supplementary material for: Population-based mechanistic modeling allows for quantitative predictions of drug responses across cell types
Source: NPJ Syst Biol Appl. 2018 Feb 24;4:11. doi: 10.1038/s41540-018-0047-2 (PMC5825396; doi:10.1038/s41540-018-0047-2)
Supplement: Supplementary file 3 — Supplemental Tables [file 41540_2018_47_MOESM3_ESM.pdf]

SUPPLEMENTARY TABLES

Content in SI	Relevance
Table S1, Table S2	Mathematical models and related model parameters
Table S3, Table S4a-b	Demonstration of cross-cell type regression model predictive strength
Table S5	Parameters modified for Heart Failure phenotype

For consistency throughout the manuscript, parameters controlling current/flux magnitude for ion channels are termed ***G***, those that control pumps and transporters are termed ***K***. Detailed definitions of all parameters are listed in Tables S1-S2, with notes explaining specific cases.

Table S1.: Parameters common in O'Hara et al. (human adult myocyte) [1] and Paci et al. (human iPSC-CM) [2] models that control ionic currents and fluxes.

Parameter	Definition	Baseline value in O'Hara et al.	Baseline value in Paci et al.
G_{Na}	Fast Na^+ maximal conductance	75 mS/μF	3.6712302e3 S/F
G_{K1}	Inward rectifier K^+ scaling factor[a]	0.1908 mS/μF	28.1492 S/F
G_{Kr}	Rapid delayed rectifier K^+ scaling factor[a]	0.046 mS/μF	29.8667 S/F
G_{Ks}	Slow delayed rectifier K^+ scaling factor[b]	0.0034 mS/μF	2.041 S/F
G_{to}	Transient outward K^+ maximal conductance	0.02 mS/μF	29.9038 S/F
G_{CaL}	L-type Ca^{2+} current permeability[c]	0.0001 cm/s	8.635702e-2 m^3/(Fxs)
G_{bNa}	Background Na^+ permeability/conductance[d]	3.75e-10 cm/s	0.9 S/F
G_{bCa}	Background Ca^{2+} permeability/conductance[d]	2.5e-8 cm/s	0.69264 S/F
K_{NCX}	Maximal Na^+-Ca^{2+} exchanger current[e]	0.0008 μA/μF	4900 A/F
K_{NaK}	Scales/Maximal Na^+-K^+ pump current[e]	30	1.841424 A/F
K_{pCa}	Maximal sarcolemmal Ca^{2+} pump current[e]	0.0005 A/F	0.4125 A/F
K_{RyR}	SR Ca^{2+} release scaling factor[f]	1.00	1.00
K_{SERCA}	SR Ca^{2+} uptake scaling factor /maximal flux[g]	1.00	0.56064 mM/s

[a]The scaling factors for I_{K1} and I_{Kr} are not formally maximal conductance, since each is multiplied by $\sqrt{K_o/5.4}$ and can therefore be greater than this value. Changing this factor scales the current at all values of extracellular $[K^+]$ while maintaining the dependence on this variable.

[b]The scaling factor for I_{Ks} is multiplied by a function of intracellular $[Ca^{2+}]$. This value is therefore not precisely the current's maximal conductance.

[c]L-type Ca^{2+} current in O'Hara et al. and Paci et al. models is computed using Goldman-Hodgkin-Katz current equation, current magnitude is therefore determined by permeability, in units of distance/time.

[d]Background Na^+ and background Ca^{2+} currents in O'Hara et al. model are computed using Goldman-Hodgkin-Katz current equation, with permeability (distance/time) controlling current magnitude; while in the Paci et al. model, these currents are computed using an Ohm's law approximation, with maximal conductance controlling current magnitude.

[e]Parameters controlling the magnitudes of Na^{+}-Ca^{2+} exchanger current, Na^{+}-K^{+} pump current and sarcolemmal Ca^{2+} pump current are expressed as maximal current densities in O'Hara et al. and in Paci et al. models.

[f]Parameters controlling the magnitude of SR Ca^{2+} release through the ryanodine receptor (RyR) in both O'Hara et al. and Paci et al. models are an introduced unitless multiplier with baseline value equals to 1.00. Changing this multiplier serves to scale the flux through this pathway.

[g]Parameter controlling the magnitude of SR Ca^{2+} uptake through SERCA in O'Hara et al. model is an introduced unitless multiplier with baseline value equals to 1.00; in Paci et al. model it is the maximal uptake rate in unit mM/s.

Table S2.: Parameters common in Livshitz et al. (guinea pig adult myocyte) [3] and Shannon et al. (rabbit ventricular myocyte) [4] models that control ionic currents and fluxes.

Parameter	Definition	Baseline value in Livshitz et al.	Baseline value in Shannon et al.
G_{Na}	Fast Na^+ maximal conductance	16 mS/μF	16 mS/μF
G_{K1}	Inward rectifier K^+ scaling factor[a]	0.75 mS/μF	0.9
G_{Kr}	Rapid delayed rectifier K^+ scaling factor[a]	0.02614 mS/μF	0.03
G_{Ks}	Slow delayed rectifier K^+ scaling factor[b]	0.433	0.07
G_{CaL}	L-type Ca^{2+} current permeability[c]	5.4e-4 cm/s	5.4e-4 cm/s
G_{bNa}	Background Na^+ conductance	0.004 mS/μF	2.97e-4 mS/μF
G_{bCa}	Background Ca^{2+} conductance	3.016e-3 mS/μF	2.513e-4 mS/μF
K_{NCX}	Na^+-Ca^{2+} exchanger current scaling factor[d]	2.5e-4	9.0 A/F
K_{NaK}	Maximal Na^+-K^+ pump current[e]	2.25 μA/μF	1.91 μA/μF
K_{pCa}	Maximal sarcolemmal Ca^{2+} pump current[e]	1.15 μA/μF	0.0673 μA/μF
K_{RyR}	SR Ca^{2+} release scaling factor[f]	0.125	25 1/ms
K_{SERCA}	SR Ca^{2+} uptake (SERCA) scaling factor[g]	8.75e-3 mM/ms	2.86e-4 mM/ms

[a]The scaling factors for I_{K1} and I_{Kr} are not formally maximal conductance, since each is multiplied by $\sqrt{K_o/5.4}$ and can therefore be greater than this value. Changing this factor scales the current at all values of extracellular [K^+] while maintaining the dependence on this variable.

[b]The scaling factor for I_{Ks} is multiplied by a function of intracellular [Ca^{2+}]. This value is therefore not precisely the current's maximal conductance.

[c]L-type Ca^{2+} current in Livshitz al. and Shannon et al. models is computed using Goldman-Hodgkin-Katz current equation, current magnitude is therefore determined by permeability, in units of distance/time.

[d]The scaling factor for the Na^+-Ca^{2+} exchange current is termed c1 in the Livshitz et al paper and is unitless.; for Shannon et al. model it is the maximal Na^+-Ca^{2+} exchange current density in unit A/F.

[e]Parameters controlling the magnitudes of Na^+-K^+ pump current and sarcolemmal Ca^{2+} pump current are expressed as maximal current densities (units of A/F) in Livshitz al. and Shannon et al. models.

[f]Scaling factor controlling the magnitude of SR Ca^{2+} release through Ryanodine receptor (RyR) is termed α_{rel} in Livshitz et al. model; it is termed ks in Shannon et al. model.

[g]Parameter controlling the magnitude of SR Ca^{2+} uptake through SERCA in Livshitz et al. and Shannon et al. models is the maximal uptake rate in units of mM/s.

Table S3.: Quantified values of 11 metrics of AP and CaT for adult myocyte under baseline, or I_{Kr}/I_{CaL} 50% blockade simulation (actual response) and the predicted ion channel blockade effects from cross-cell type regression (predicted response).

		**I_{Kr} 50% block**			**I_{CaL} 50% block**		
	Baseline	Actual response	Predicted response	**Accuracy %[a]**	Actual response	Predicted response	**Accuracy %[a]**
APD (ms)	256	370	363	**98.2**	218	211	**96.7**
APD_{50} (ms)	192	260	260	**99.9**	154	152	**98.8**
APD_{90} (ms)	246	352	347	**98.7**	206	201	**97.9**
V_{peak} (mV)	40.2	40.6	41.0	**101**	38.0	37.92	**99.8**
V_{rest} (mV)	-88.0	-88.0	-88.0	**100**	-88.0	-88.0	**100**
CaTA (µM)	0.272	0.311	0.319	**102**	0.101	0.106	**105**
CaD_{50} (ms)	230	225	226	**100**	317	299	**94.6**
CaD_{90} (ms)	573	577	567	**98.2**	679	666	**98.1**
Ca_{peak} (µM)	0.357	0.399	0.405	**102**	0.174	0.178	**102**
Ca_{rest} (µM)	0.0852	0.0875	0.0878	**100**	0.0730	0.0722	**98.8**
Decay time (ms)	256	265	259	**97.6**	291	289	**99.1**

[a]Percentage accuracy was calculated for individual metric as

$$\frac{Predicted\ Response}{Actual\ Response} * 100\%$$

Results are rounded to 3 significant digits.

Table S4.: Across 10 ion transport pathways, 50% blockade of an individual pathway resulted in adult myocyte APD_{90} (a) and CaTA (b) changes relative to baseline values were quantified (actual response); these changes were also predicted with cross-cell type regression (cross-cell type predicted) and directly approximated from spontaneous contracting iPSC-CM behavior (spontaneous approximated). Results from 100-cell population are represented as percentage (%) mean ± standard deviation.

Table S4a

	APD_{90} (% change)					
current/ flux	Actual response	Cross-cell type predicted	**Prediction residual inaccuracy[a]**	Spontaneous approximated	**Spontaneous residual inaccuracy[a]**	**% Improve[b]**
I_{Na}	3.72±2.62	5.42±4.34	1.70	17.0±3.91	13.3	87.2
I_{K1}	0.863±0.458	4.91±4.77	4.04	46.1±14.1	45.3	91.1
I_{Kr}	46.4±4.31	43.3±6.26	3.11	35.2±5.30	11.2	72.1
I_{Ks}	3.14±0.766	1.40±1.20	1.73	0.554±0.23	2.58	N/A[c]
I_{to}	-0.249±0.503	2.88±2.14	3.13	14.8±3.89	15.1	79.3
I_{CaL}	-13.8±2.07	-15.7±7.81	1.93	-37.8±7.48	24.0	92.0
I_{NCX}	-6.02±1.69	-3.54±6.64	2.48	-12.4±5.09	6.43	61.4
I_{NaK}	2.53±0.951	1.43±3.51	1.10	-2.20±9.83	4.73	N/A[c]
J_{RyR}	-1.66±1.10	0.337±4.75	2.00	-5.04±4.13	3.38	N/A[c]
J_{SERCA}	0.0424±3.04	-3.17±5.04	3.21	6.75±16.7	6.70	52.1

Table S4b

	CaTA (% change)					
current/ flux	Actual response	Cross-cell type predicted	**Prediction residual inaccuracy[a]**	Spontaneous approximated	**Spontaneous residual inaccuracy[a]**	**% Improve[b]**
I_{Na}	40.5±14.1	44.2±5.21	3.69	8.76±7.09	31.8	88.4
I_{K1}	-0.482±0.263	9.35±8.95	9.83	0.581±4.32	1.06	N/A[c]
I_{Kr}	14.5±6.24	19.2±6.37	4.66	5.01±3.99	9.51	51.0
I_{Ks}	2.05±0.641	0.968±1.65	1.09	0.075±0.0605	1.98	N/A[c]
I_{to}	-5.78±1.57	-0.0282±4.02	5.75	6.71±4.32	12.5	54.0
I_{CaL}	-58.6±6.95	-57.4±5.32	1.12	-44.4±5.66	14.1	92.1
I_{NCX}	132±22.9	129±20.1	2.70	9.70±8.46	122	97.8
I_{NaK}	19.8±3.66	16.1±6.26	3.70	30.3±21.4	10.5	64.7
J_{RyR}	-19.1±6.16	-13.5±6.42	5.58	-15.9±7.16	3.21	N/A[c]
J_{SERCA}	-63.9±9.09	-70.4±3.60	6.47	9.45±27.5	73.4	91.2

[a] Residual inaccuracy for cross-cell type predictions or approximation from iPSC-CM spontaneous behavior relative to adult myocyte responses simulated with adult model was calculated as

$$|Cross\ cell\ type\ predicted\ Response - Actual\ Response|$$

Or

$$|Spontaneous\ approximated\ Response - Actual\ Response|$$

[b] Percentage improvement was calculated as

$$\frac{|Prediction\ Residual\ Inaccuracy - Spontaneous\ Residual\ Inaccuracy|}{Spontaneous\ Residual\ Inaccuracy} * 100\%$$

Representing the correction of mismatch achieved with cross-cell type regression method.
Results are rounded to 3 significant digits.

[c] For some ion transport pathways, responses seen in adult myocyte and iPSC-CM under spontaneous contraction are fairly close (spontaneous residual inaccuracy < 5%), therefore percentage improvement with cross-cell type predictions was not computed.

Table S5.: Ionic parameters modified according to prior work [5] to reproduce heart failure (HF) phenotype in O'Hara et al. human adult myocyte model .

Parameter	Definition	% in the HF compared to baseline model
I_{NaL}	Late Na^+ current	180%
tau_{hL}	Time constant for late Na^+ channel inactivation	180%
I_{to}	Transient outward K^+ current	40%
I_{K1}	Inward rectifier K^+ current	68%
I_{NaK}	Na^+-K^+ pump current	70%
I_{NCX}	Na^+-Ca^{2+} exchanger current	175%
J_{SERCA}	SR Ca^{2+} uptake flux via SERCA	50%
I_{leak}	SR Ca^{2+} leak flux	130%
$J_{RyR,\ NP,\ Ca\ sensitivity}$	SR Ca^{2+} release flux via RyR, non- phosphorylated RyR sensitivity to Ca^{2+}	80%
CaMKa	Ca^{2+} calmodulin-dependent protein kinase II	150%

References

1-O'Hara, T., Virag, L., Varro, A. & Rudy, Y. Simulation of the undiseased human cardiac ventricular action potential: model formulation and experimental validation. *PLoS Comput Biol* **7**, e1002061 (2011).

2-Paci, M., Hyttinen, J., Aalto-Setala, K. & Severi, S. Computational models of ventricular- and atrial-like human induced pluripotent stem cell derived cardiomyocytes. *Ann Biomed Eng* **41**, 2334-2348 (2013).

3-Livshitz, L. & Rudy, Y. Uniqueness and Stability of Action Potential Models during Rest, Pacing, and Conduction Using Problem-Solving Environment. *Biophysical Journal* **97**, 1265-1276 (2009).

4-Shannon, T. R., Wang, F., Puglisi, J., Weber, C. & Bers, D. M. A Mathematical Treatment of Integrated Ca Dynamics within the Ventricular Myocyte. *Biophysical Journal* **87**, 3351-3371 (2004).

5-Gomez, J. F., Cardona, K., Romero, L., Ferrero, J. M., Jr. & Trenor, B. Electrophysiological and structural remodeling in heart failure modulate arrhythmogenesis. 1D simulation study. *PLoS One* **9**, e106602 (2014).
